# Circular RNAs Associated with Human Cytomegalovirus Infection

**DOI:** 10.3390/molecules31183208

**Published:** 2026-09-11

**Authors:** Cristian J. Pagtalunan, Isadora Zhang, Fenyong Liu

**Affiliations:** 1 School of Public Health, University of California, Berkeley, CA 94720, USA; 2 Department of Chemical and Biomolecular Engineering, University of California, Berkeley, CA 94720, USA; 3 Program in Comparative Biochemistry, University of California, Berkeley, CA 94720, USA

**Keywords:** circular RNA, cytomegalovirus, herpesvirus, gene expression, non-coding RNAs

## Abstract

Human cytomegalovirus (HCMV) can cause life-threatening diseases in immunocompromised individuals. Moreover, HCMV is the leading cause of virus-associated congenital infections. As one of the eight human herpesviruses, HCMV is particularly associated with a range of neurological impairments, including microcephaly, hearing loss, and developmental delays. Given its significant impact on early development, there is strong motivation to explore alternatives and complements to current antiviral therapies to better mitigate HCMV-associated diseases. Like other herpesviruses, HCMV establishes lifelong infections characterized by alternating latent and lytic phases, enabling the virus to evade immune clearance. These transitions are accompanied by substantial changes in viral and host gene expression, which influence cellular pathways and regulatory mechanisms. Circular RNAs (circRNAs) have recently been identified as products of the HCMV transcriptome and have attracted increasing attention due to their potential roles in infection and pathogenesis. This review focuses on elucidating the functions of HCMV-derived circRNAs, their interactions with both viral and host transcriptomes, and the methodologies used for their identification and analysis. Recent advances in this field have expanded our understanding of the HCMV transcriptome and its contribution to disease, highlighting circRNAs as potential candidates for future biomarker or therapeutic studies.

## 1. Introduction

HCMV, also known as human herpesvirus 5 (HHV-5), belongs to the β-herpesvirus subgroup within the herpesvirus family [1,2]. It shares important biological characteristics with other herpesviruses, including the ability to establish lifelong latent infections and reactivate under favorable conditions [3]. Understanding the HCMV life cycle and developing new strategies to treat this virus are crucial, particularly given its significant impact on immunocompromised individuals and its role as a leading cause of congenital disease [4], for which effective preventive measures remain limited. Recently, virally encoded circular RNAs (circRNAs) have garnered increasing attention, and their roles in viral infection and disease progression have become key areas of focus in research [5,6]. Examining the specific functions of HCMV circRNAs and exploring how they are identified and analyzed will be the primary purpose of this review. The aim of this work is to summarize recent advances in circRNA research and discuss how these findings enhance the understanding of the HCMV transcriptome. By contributing to the growing body of knowledge surrounding HCMV circRNAs, this review shall support the development of novel antiviral strategies targeting these molecules.

## 2. Human Cytomegalovirus (HCMV)

Human cytomegalovirus (HCMV) affects up to 85% of the population, establishes lifelong infection in humans, and exhibits a significant clinical impact in immuno-compromised populations [2,7]. Unlike Kaposi’s sarcoma-associated herpesvirus (KSHV) and the Epstein–Barr virus (EBV), which are other human herpesviruses, HCMV is not explicitly classified as oncogenic; instead, previous research suggests it participates in oncomodulation, meaning that it exacerbates tumor progression and immune evasion in certain cancers [8]. HCMV is a leading cause of congenital infections worldwide and is associated with severe complications including sensorineural hearing loss [9], neurodevelopmental delays [10], and, in extreme cases, infant mortality demise [11]. Typically, HCMV infection in healthy individuals is met with no major symptoms apart from infectious mononucleosis [12]. In immunocompromised individuals, such as transplant recipients, patients with HIV, and patients undergoing chemotherapy, HCMV infection can lead to life-threatening diseases including pneumonitis, retinitis, colitis, and systemic viremia [12,13].

Transmission of HCMV most often occurs through direct contact between mucosal membranes and bodily fluids from infected individuals, regardless of whether the virus is in a dormant or active state [14]. Vertical placental transmission from the mother to the fetus during pregnancy represents a major route of congenital infection, but other routes such as organ and stem cell transplantation are also potential modes of general infection [15]. Seroprevalence of HCMV infection can widely vary across adult populations depending on geographic location, socioeconomic status, and public health risk factors; in European countries, seroprevalence rates of HCMV infection are generally lower than those observed in the Middle East and Africa [16]. As with KSHV, disease severity is strongly influenced by the host immune status, with immunocompetent individuals typically experiencing asymptomatic or mild infections, while immunocompromised patients are at significantly higher risk for severe disease [13]. In fact, HCMV is the most common opportunistic infection for individuals who are simultaneously diagnosed with acquired immunodeficiency syndrome (AIDS), accounting for up to 40% of disease presentation among patients [2,17].

Current treatment strategies for HCMV infection depend on the clinical context and patient risk factors. First-line antiviral therapies include nucleoside analogs (e.g., ganciclovir and valganciclovir), while second-line measures consist of both nucleotide analogs (e.g., cidofovir) and inorganic pyrophosphate analogs (e.g., foscarnet); both the first-line and second-line therapies target the viral DNA polymerase to inhibit replication of viral genes, but the second-line therapies are often utilized in refractory cases [18]. More recently, newer agents such as letermovir have been developed for prophylaxis in transplant patients receiving hematopoietic stem cells [19]. Despite these advances, antiviral resistance, drug toxicity, and the inability to fully eradicate latent virus remain significant challenges. No universally approved vaccine for HCMV is currently available, though several candidates are under active investigation.

**Figure 1:**
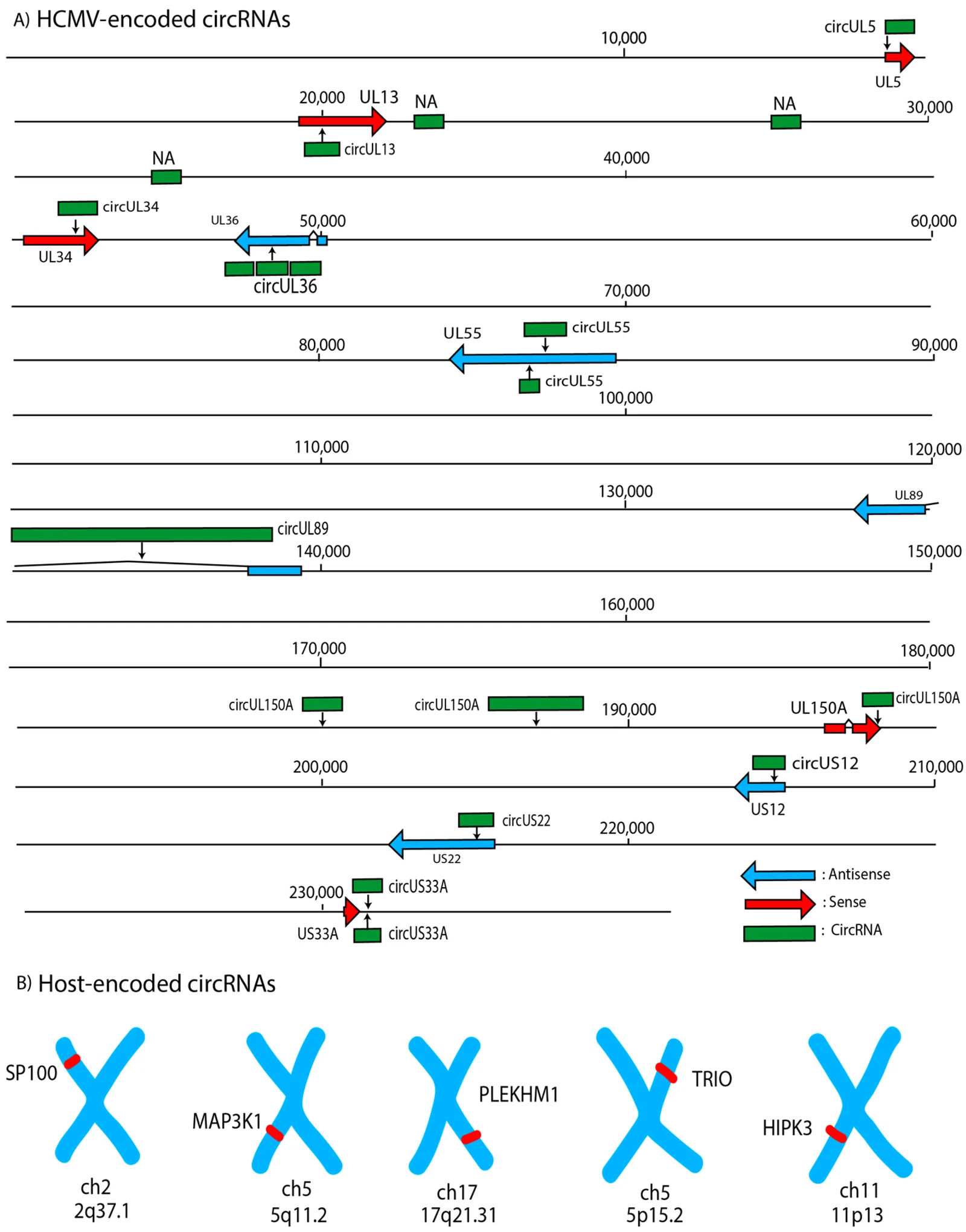
The top twenty most encoded circRNA transcripts within the HCMV genome [6] and the five parent genes of associated upregulated human circRNAs during HCMV infection discussed within this review. (**A**) Mapping of known HCMV genes alongside researched circRNAs that span certain loci in the HCMV genome; reading frames are separated by color to distinguish the direction of transcription, whereas circRNA are denoted by green boxes superimposed upon the original parent gene. (**B**) Annotated chromosome locations of the upregulated human circRNAs and the genes that they occupy.

Human cytomegalovirus possesses the largest double-stranded linear DNA genome among human herpesviruses, measuring approximately 235 kb and encoding over 200 open reading frames that contribute to viral replication, immune evasion, and host modulation (Figure 1) [20]. Additionally, the transcriptome is further regulated by various types of RNAs, which include messenger RNAs (mRNAs), long non-coding RNAs (lncRNAs), microRNAs (miRNAs), and circular RNAs (circRNAs) [21]. Although circRNAs are the main purpose of the review, they are heavily associated with their parent genes as certain splicing schemes dictate whether the linear or circular transcript is made (Figure 1).

The virion structure consists of an icosahedral capsid, a surrounding tegument layer rich in regulatory proteins, and a lipid envelope studded with glycoproteins essential for host cell entry [22]. Like other herpesviruses, the HCMV life cycle consists of the transition between two phases—the dormant latent stage and active lytic replication [23]. During latency, the viral genome persists primarily in host cells such as hematopoietic progenitor cells and monocytes, where the virus exhibits minimal gene expression and produces few to no infectious virions [24]. Reactivation from latency into the lytic phase can occur in response to cellular differentiation, inflammation, or immunosuppression [25]. Epigenetic mechanisms, including chromosomal modifications, contribute to regulating viral progression and influence whether the virus remains in a latent state or transitions to the lytic stage [23]. The lytic cycle is characterized by a tightly regulated cascade of immediate-early (IE), early (E), and late (L) gene expression; immediate-early genes, particularly IE1 and IE2, act as key transcriptional regulators that initiate the viral replication program [26]. Cellular signaling pathways initiated by activation of inflammatory IL-6, including MAPK/ERK and SFK signaling, play critical roles in promoting viral reactivation and gene expression [27]. Once activated, these pathways facilitate viral DNA replication, structural protein synthesis, and eventual assembly and release of progeny virions, enabling further dissemination within the host [28].

## 3. General circRNA Expression Across Hosts and Herpesviridae

Multiple classes of non-coding RNAs are expressed during the human cytomegalovirus (HCMV) lifecycle, contributing to the virus’s complex regulatory network and influencing host gene transcription [29]. Among these, the relatively novel class of circular RNAs (circRNAs) has garnered increasing attention, necessitating investigation into their implications in modulating viral infection. For context, circRNAs are generally classified, with some exceptions, as non-coding due to their lack of a 5′ cap and 3′ polyadenylated tail, features that are typically required for canonical translation initiation [30]. Additionally, previous studies have demonstrated through polysome gradient analysis and ribosome profiling that circRNAs are not actively associated with polysomes [31]. However, circRNA research also confirmed that a subset of circRNAs possess protein-coding potential; for example, circ-ZNF609, a circRNA expressed in murine and human myoblasts, has an open reading frame that can be translated in either a splice-dependent or cap-independent manner [32,33]. Despite their typical classification as non-coding, these transcriptional products are suspected to contribute, to some extent, to the overall pathogenesis of certain diseases. Particularly, circRNAs derived from the HCMV genome have become an emerging area of interest due to their potential involvement in multiple layers of viral and host regulation [34]. In other DNA herpesviruses, such as KSHV and EBV, certain circRNAs are known to participate in coordinated regulatory networks that shape the viral transcriptome and exert reciprocal effects, such as the promotion of viral reactivation or tumorigenic environments, on host regulatory pathways [35,36]. Early studies of viral circRNAs began with their discovery in the hepatitis delta virus, and subsequent work has established that circRNA expression is widespread [37]. While circRNAs were initially characterized in eukaryotic systems, their identification has since expanded across a wide range of organisms, especially DNA viruses including human herpesviruses.

In general, circRNAs are implicated in diverse biological functions, including regulating gene expression, sequestering microRNAs and proteins, and acting as scaffolding molecules that facilitate protein–protein interactions [37,38,39]. The ability of circRNAs to function as molecular sponges enables them to bind and sequester regulatory microRNAs or proteins, preventing these factors from interacting with their endogenous targets and thereby modulating downstream gene expression [40]. Additionally, circRNAs can serve as structural platforms, i.e., protein scaffolds, that bring multiple proteins into proximity, influencing the assembly or activity of functional complexes [41]. Collectively, these properties are particularly relevant in HCMV infection, where host-derived circRNAs that are differentially expressed upon infection may interact with complementary proteins and affect post-transcriptional regulatory processes [34]. For example, across certain herpesvirus transcriptomes such as KSHV, EBV, and HCMV, lytic infection can modulate host regulatory processes by upregulating a specific human circRNA, i.e., hsa_circ_001400, which eventually inhibits apoptotic mechanisms via the PI3K–AKT–mTOR signaling pathway [42].

These findings regarding shared transcript upregulation across DNA herpesviruses may also suggest conservation, as previous studies have concluded that circRNA-related features are more likely to be conserved among DNA viruses, resulting in greater sequence similarity compared to RNA viruses [43]. In fact, for the viral circRNAs in RNA viruses that do not share the same degree of conservation as other DNA viruses, research suggests that the circularization mechanisms have evolved more recently [43,44]. However, overall knowledge concerning the specific circRNAs encoded by HCMV and whether they exhibit sequence conservation with related viruses is insufficient to conclude exact conservation with a common ancestor. This observation is consistent with broader findings that viral circRNAs are generally less evolutionarily conserved than host-derived circRNAs, although functional convergence may still occur at the level of regulatory pathways [44]. Nonetheless, the presence of circRNAs across diverse herpesviruses highlights their potential importance in viral biology and warrants further investigation into their evolutionary and functional significance in HCMV.

Research has demonstrated that circRNAs are now recognized as functional regulatory elements with potential implications in disease processes, including viral infections [37]. In the context of HCMV, ongoing studies aimed at manipulating circRNA expression, examining their expression profiles, investigating behavior during lytic and latent phases, and profiling associated transcriptomic changes may provide insight into their roles in viral replication and host interaction. Such approaches may also support the development of circRNA-based strategies as potential therapeutic tools for controlling HCMV infection.

## 4. Biogenesis of circRNAs Transcribed by Viral Genomes

**Figure 2:**
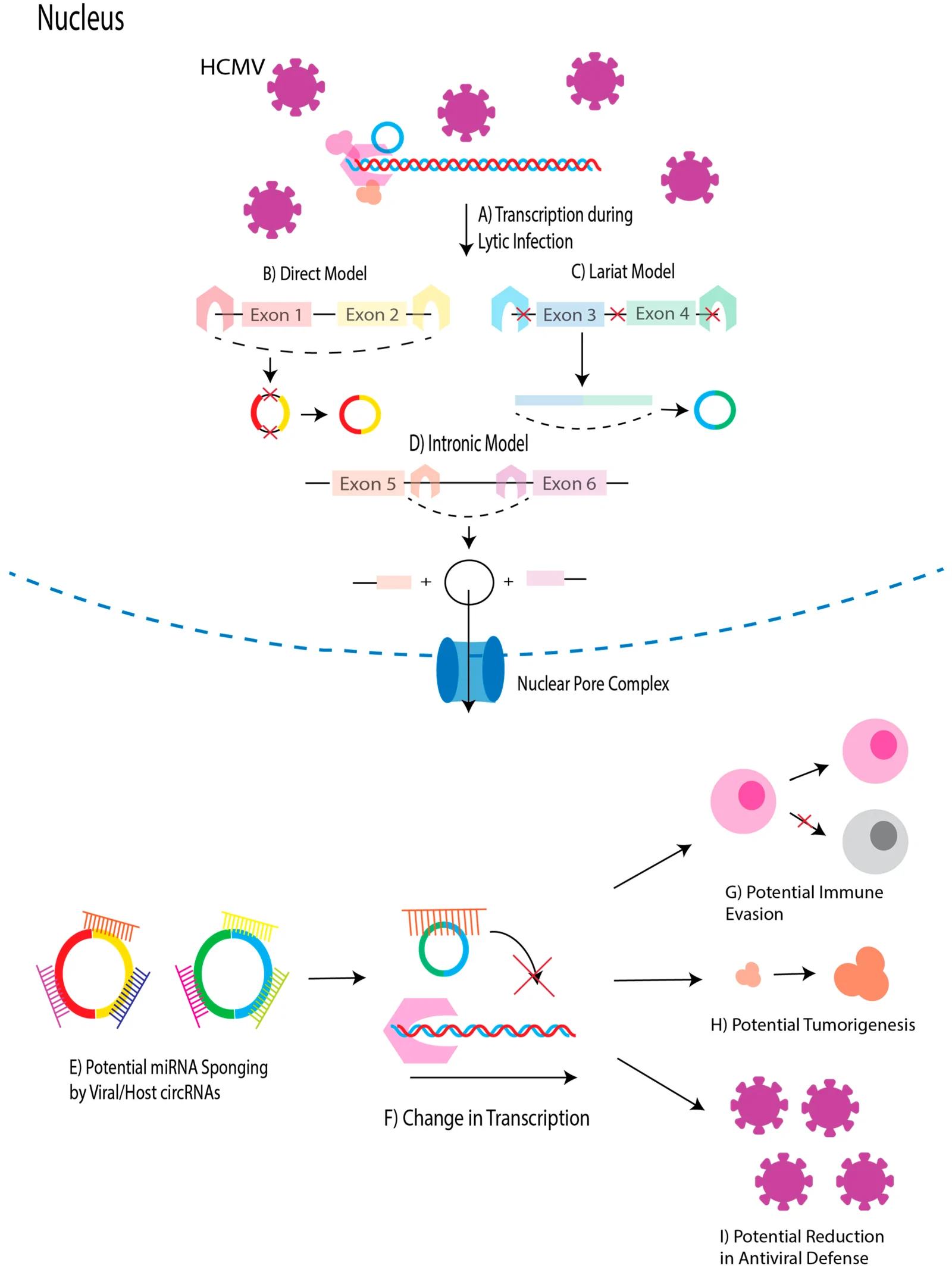
Overview of circRNA biogenesis in the context of HCMV infection, predicted circRNA functions, and hypothesized impacts. (**A**) HCMV infection drives active viral genomic transcription within the host cell nucleus, producing primary pre-mRNA transcripts. (**B**) In the direct back-splicing model, intronic complementary sequences or protein factors bring flanking splice sites into proximity on nascent pre-mRNA, facilitating back-splicing prior to or concurrently with canonical splicing. (**C**) In the lariat-driven model, exon skipping during canonical splicing generates an exon-containing lariat intermediate, which subsequently undergoes back-splicing to yield a mature circular RNA (circRNA) (**D**) In the intronic circularization model, selected excised introns escape degradation and are circularized to form stable circular intronic RNAs (ciRNAs). (**E**) Functions of HCMV-associated circRNAs primarily include the sequestration of microRNAs (miRNA sponging), which is commonly predicted amongst HCMV circRNA studies. (**F**) By sequestering miRNAs, HCMV circRNAs may prevent miRNA-mediated degradation or translational inhibition of target mRNA transcripts, driving downstream gene regulatory changes. (**G**) Altered signaling pathways may impair host immune cell recognition and apoptotic responses. (**H**) Downstream gene expression changes may also promote cell proliferation/oncogenesis. (**I**) Increased viral replication may also result from a reduction in antiviral defense due to influence of differential circRNA expression.

HCMV-derived circRNAs are thought to follow mechanisms analogous to those described in other DNA viruses since studies have confirmed their presence in other human herpesviruses [35]. Previous bioinformatic analyses have identified circRNAs originating from multiple regions of the HCMV genome, suggesting that circularization is a widespread feature of viral gene expression during the lytic phase (Figure 1) [6]. Proposed models of circRNA biogenesis include a form of alternative splicing known as back-splicing, in which a downstream 5′ splice donor site is covalently linked to an upstream 3′ splice acceptor site, forming a closed circular RNA molecule via a phosphodiester bond (Figure 2) [45]. The resulting junction, termed the back-splice junction (BSJ), serves as a defining feature for circRNA identification [46]. Numerous computational and experimental initiatives aimed at systematically identifying BSJ sites across genomes represent critical initial steps in circRNA research, facilitating the subsequent characterization of their functional roles.

Two primary models have been proposed to explain general circRNA formation: the lariat-driven model and the direct back-splicing model (Figure 2) [47]. In the direct back-splicing model, base-pairing between flanking intronic complementary sequences or interactions among trans-acting RNA-binding proteins bring a downstream splice donor and an upstream splice acceptor into close proximity on nascent pre-mRNA, allowing back-splicing to occur prior to or concurrently with canonical splicing. Conversely, in the lariat-driven model, canonical splicing events such as exon skipping generate an exon-containing lariat intermediate, which subsequently undergoes internal back-splicing to yield mature circular RNA species. In both scenarios, additional processing steps remove residual intronic sequences, yielding mature circRNAs capable of participating in regulatory functions (Figure 2). Notably, alternative splicing events, including exon skipping, may give rise to circRNAs that retain intronic regions, thereby contributing to transcriptomic diversity. The commitment of potential circRNA candidates to back-splicing is governed by the interplay of trans-acting factors and cis-regulatory elements, which collectively influence the efficiency and specificity of circRNA biogenesis (Figure 2) [48]. Host-derived splicing machinery, including spliceosomal small nuclear ribonucleoproteins (snRNPs), plays a central role in mediating both canonical and back-splicing events [49]. Additional trans-acting factors, such as RNA helicases and RNA-binding proteins (RBPs), facilitate circularization by modulating the RNA secondary structure and promoting proximity between splice donor and acceptor sites [50]. The diverse mechanisms of circRNA biogenesis contribute to the molecular properties of circRNAs, which in turn determine their functional roles as miRNA sponges, protein scaffolds, regulators of RNA-binding proteins, and modulators of gene transcription (Figure 2).

Although viral proteins directly analogous to those implicated in circRNA biogenesis in other DNA herpesviruses have not been definitively identified in HCMV, HCMV infection has been shown to alter host circRNA expression, indicating that immediate-early viral gene products may play a role in regulating circRNA biogenesis [6]. Cis-regulatory elements within both the viral and host genomes further contribute to circRNA formation. Intronic complementary sequences, including inverted repeats and other regions of sequence homology, can promote base pairing that brings flanking splice sites into proximity, thereby enhancing the likelihood of back-splicing [51]. In fact, one previous study utilizing WebLogo revealed that fourteen analyzed HCMV-derived circRNAs exhibit a canonical “AGN|UNN” motif at the acceptor site and a noncanonical “NAG|GUN” motif at the donor site, suggesting that there is a prominence for recognizable flanking sequences that regulate circRNA biogenesis [5].

## 5. Methodologies Utilized in HCMV circRNA Research

Robust characterization of HCMV and host circRNAs depends on an integrated framework combining computational discovery with rigorous orthogonal experimental validation. To overcome the limitations of short-read bioinformatic predictions, confirmation requires multiple complementary testing modes. As outlined by a previous review regarding best practices in circRNA research, identification methods primarily include collecting an initial pool of RNA from treated in vitro models, RNase R resistance assays with linear controls, BSJ verification via divergent RT-PCR and Sanger sequencing, exclusion of genomic DNA artifacts, and structural resolution via Northern blot or long-read sequencing platforms [52].

Various in vitro models have been utilized to study HCMV dynamics, including primary human fibroblasts, epithelial cells, and endothelial cells, all of which represent physiologically relevant targets of infection. Specifically, human leukemia monocytes (THP-1) have previously been used to examine circRNA transcriptional responses to HCMV infection [53]. Studies involving primary fibroblasts (HFFs), endothelial cells (ECs), neural progenitors (NPCs) that originated from embryonic stem cells (ESCs), and human embryonic lung fibroblasts (HELFs) were also rigorously analyzed for BSJ-containing sites, a defining feature of circRNAs that contributes to the identification and quantification of circRNA expression across the genome [5]. Although cell models are incredibly useful in examining how the transcriptome changes over time, the potential physiological effects of the surrounding tissue prevent holistic conclusions from being made. Despite these limitations, findings derived from such models provide strong support for the biological relevance of circRNAs in HCMV pathogenesis as models attempt to capture in vivo infection contexts. However, further studies in additional in vivo models capable of supporting HCMV infection are needed to ensure consistent effects on the relative expression levels of both host and viral circRNAs.

Enrichment strategies, such as RNase R treatment, are commonly employed prior to sequencing to degrade linear RNA transcripts and improve the detection of circular transcripts, which are resistant to exonuclease activity due to their covalently closed structure [54]. However, it should be noted that highly structured linear RNAs, such as those containing G-quadruplexes, can resist RNase R degradation, whereas excessive digestion or endonuclease contamination risks degrading true circular transcripts. To ensure experimental accuracy, researchers must always run parallel mock-treated controls and evaluate digestion efficiency by quantifying the depletion of highly expressed housekeeping genes like GAPDH and ACTB [55]. RNA-Seq analyses comparing latently infected and mock-infected cell lines in these systems have demonstrated that HCMV infection alters both viral and host circRNA expression profiles, with evidence of circRNA upregulation and downregulation, thereby confirming a measurable impact on the transcriptome [56]. Integration of RNA-Seq data with quantitative validation methods such as qRT-PCR has further strengthened observed associations of circRNAs in various cancer cell lines [57]; therefore, the incorporation of these approaches can enable quantitative assessment of how specific transcripts are altered in response to HCMV infection and how host circRNA landscapes are subsequently modulated.

Further analyses involving divergent primers, Sanger Sequencing, and Northern blot are common techniques utilized to verify the transcription of potential circRNA candidates [6]. When a transcript undergoes back-splicing to form a closed circle, the downstream splice donor fuses to an upstream splice acceptor. This head-to-tail circularization brings the outward-facing divergent primers into a convergent orientation spanning the BSJ, allowing selective RT-PCR amplification of the circular isoform while ignoring the linear parental mRNA [58]. Although divergent primers are designed to selectively amplify circular structures, they can also inadvertently amplify template-switching artifacts, trans-spliced linear RNAs, or tandem genomic duplications. To rule out genomic DNA-derived false positives, parallel PCR must be performed on genomic DNA (gDNA) controls alongside Sanger sequencing of the complete back-splice junction (BSJ) product.

Bioinformatic approaches that investigate viral transcriptomes in infected cells are central to the identification and characterization of HCMV-derived circRNAs. A growing number of studies have applied diverse computational pipelines to map back-splice junction (BSJ)-containing reads and determine the genomic origins of HCMV circRNAs [6]. Bioinformatic detection of BSJs frequently relies on complementary algorithms such as CIRI2, which scans BWA-MEM alignment files for split-mapped reads, and find_circ, which isolates unmapped reads and realigns terminal 20-nucleotide anchors via Bowtie2 [40,59,60]. Such algorithms are frequently used to identify BSJ-spanning reads and predict circRNA candidates in HCMV research [5]. Because each tool employs distinct alignment logic, requiring candidate circRNAs to be called by both pipelines significantly reduces computational false positives [52]. Concerns over false positives have also led to the construction of refined algorithms, like CIRIquant, which incorporate additional filtering and validation steps to improve accuracy [61]. Furthermore, candidates must satisfy strict read-depth filtering (typically ≥ 2 unique BSJ reads) to eliminate background noise. More advanced frameworks incorporate quantitative metrics, such as junction read counts, to assess circRNA abundance and reliability. Therefore, to enable valid comparisons across independent HCMV datasets, circRNA expression should be reported using normalized metrics, such as BSJ reads per million (RPM) mapped reads, or expressed as a circular-to-linear ratio against the parental mRNA, rather than relying on uncorrected absolute read counts. These sequences are subsequently aligned to reference genomes to identify unmapped reads, which may serve as potential candidates for circRNA analysis [6].

Other algorithms, such as Ularcirc, a bioinformatics tool designed to identify, visualize, and analyze circular RNAs (circRNAs) by integrating back-splice junction (BSJ) and forward splice junction (FSJ) evidence with information on internal exon usage from RNA-Seq data, can further support efforts to accurately identify circRNAs [46]. These pipelines largely represent the bioinformatic toolkit involved in broader circRNA identification, but other pipelines can extend into prediction of potential interacting factors. Previous HCMV research utilized the miRanda software (v3.3a) to predict binding interactions between miRNAs and related targets [6]. Incorporation of these techniques facilitates efforts dedicated to regulatory impacts of novel circRNAs in HCMV and related DNA herpesviruses.

Despite these advances, short-read sequencing technologies remain limited in their ability to resolve full-length circRNA isoforms and exon composition. Northern blotting has traditionally provided an essential, gel-based validation step to directly confirm total transcript size and verify full-length circular isoforms without assuming canonical exonic structures. Additionally, recent progress in long-read sequencing platforms has begun to address these limitations by enabling the direct sequencing of full-length circRNAs. Researchers have employed the isoCirc pipeline to map circRNA isoforms across multiple cell lines. By creating concatemeric long-read constructs, this strategy enables the identification of back-splice junctions (BSJs) and the assembly of full-length isoforms, resulting in a detailed molecular catalog [62]. Approaches that combine rolling-circle amplification with long-read sequencing have significantly improved isoform resolution and detection sensitivity, providing new approaches/tools to explore circRNA diversity in HCMV-infected cells [63]. Collectively, these methodological advances are enhancing the ability of researchers to characterize the complexity of circRNA expression and function within the HCMV transcriptome.

## 6. HCMV circRNAs and Their Potential Roles

To prioritize certain circRNA candidates for functional investigation, researchers often focus on transcripts that exhibit distinctive features, such as high abundance, dynamic expression changes during different stages of infection, or predicted interactions with regulatory molecules. For instance, circRNAs that are significantly upregulated during lytic infection or latency may indicate functional relevance in viral replication or persistence, as supported by studies examining THP-1 cells following viral reactivation [53]. Additionally, computational prediction of circRNA–microRNA or circRNA–protein interactions has guided efforts to define circRNA-mediated regulatory networks, particularly those that influence host antiviral responses or viral gene expression [5]. Stage-specific expression patterns further refine candidate selection, as circRNAs that are differentially expressed between latent and lytic phases may play roles in regulating viral reactivation, including in HCMV.

To date, circRNAs expressed from the HCMV genome have attracted growing attention, particularly in relation to their biogenesis, differential expression during lytic and latent phases, and potential utility as miRNA sponges [6]. Elucidating their interactions with additional cellular and viral pathways may provide further insight into their roles in modulating host–virus dynamics and disease progression. Although emerging studies suggest associations between circRNA expression and the regulation of viral genes across latent and lytic states, definitive causal relationships have yet to be established for most identified HCMV-derived circRNAs. Much of the current research on HCMV circRNAs, including their expression patterns, conservation, and potential contributions to pathogenic outcomes, remains in its early stages. Investigating the relationships between specific circRNA transcripts and the viral open reading frames (ORFs) from which they are derived or with which they overlap may yield critical insights into their mechanisms of biogenesis and functional significance within the HCMV lifecycle. Collectively, the circRNAs discussed in this review represent promising avenues for future investigation, particularly regarding their potential roles in viral pathogenesis and HCMV-associated disease progression (Table 1).

### 6.1. circUS12

One prominent HCMV circRNA identified as the most highly transcribed during lytic infection in human embryonic lung fibroblasts (HELFs) is circUS12, which is derived from the US12 gene [6]. Additionally, it should be noted that the expression levels and validation status for all HCMV circRNAs (i.e., circUS12, circUL55, and circUL89) reflect findings obtained specifically from lytic HCMV infection models in HELFs. While the linear US12 viral protein has been experimentally demonstrated to promote autophagy via ULK1 phosphorylation and LC3-II conversion [64], the specific biological functions of the circular transcript, circUS12, remain to be experimentally validated. Amplification of transcripts revealed variability in sequencing length, likely due to the presence of multiple isoforms corresponding to circUS12, suggesting that it arises from alternative splicing. Functionally, circUS12 has been predicted to target a total of 26 human cellular microRNA molecules. These target miRNAs are associated with critical cellular processes, including ion binding, protein/enzyme interactions, and transcription factor activity [6]. Hypothetically, by interfering with host miRNAs that facilitate proper protein scaffolding, circUS12 may disrupt key cellular defense mechanisms, thereby promoting efficient viral infection. However, it should be noted that direct regulatory networks involving circUS12 or its conservation across the herpesvirus family have not yet been fully defined, necessitating further characterization of its interactions with other molecular factors prior to making conclusions.

### 6.2. circUL55

Like circUS12, another validated HCMV-derived circular RNA identified to be upregulated upon entry to the lytic cycle is circUL55 [6]. Validation was achieved using RT-PCR with divergent primers, while Sanger sequencing experimentally confirmed its back-splice junction (BSJ). However, these efforts recovered only a partial sequence of circUL55, leaving the researchers to propose a rolling replication method to complete the full-length transcript in future studies [6]. Notably, circUL55 is derived from the UL55 gene, which encodes a major viral glycoprotein (gB) that serves as a critical target of host immune recognition [65]. The circRNA sequence was found to be identical to its linear UL55 transcript, which suggests that circUL55 may retain coding potential or structural features relevant to viral protein expression, but this requires further experimentation to confirm. Additionally, like circUS12, circUL55 was found to have multiple associated isoforms. Network analyses of HCMV circRNAs suggest that circUL55 possesses predicted miRNA-binding capacity, with computational models indicating potential interactions with host microRNAs involved in antiviral defense pathways [6]. One of the thirty-three notable interactions involves hsa-miR-191-5p, which has been characterized as a tumor suppressor in renal cell carcinoma and prostate cancer, while exhibiting oncogenic activity in colon, breast, and gastric cancers [66,67,68]. However, it should be noted that tumorigenesis as a response to explicit upregulation of circUL55 has not been outlined.

### 6.3. circUL89

Another studied HCMV circRNA that is highly expressed upon lytic infection is circUL89, which was among the top-ranked circRNAs during lytic infection in HELFs [6]. The transcript corresponding to circUL89 originates from the UL89 gene, a locus involved in viral genome packaging [69]. Notably, circUL89 is substantially longer than many other viral circRNAs and exists as a single transcript without evidence of alternative splicing, suggesting a more structurally conserved formation from primary versions of the RNA. Sequence analysis further revealed that circUL89 is derived from antisense transcription and includes both coding and intronic regions of the linear UL89 transcript.

Although no experimental validation has occurred, bioinformatic analyses predict that circUL89 possesses an extensive miRNA-binding capacity encompassing both host and viral microRNAs [6]. It has been hypothesized that its expression might influence native UL89 expression or modulate viral replication networks. However, these regulatory roles remain theoretical models based on genomic organization and target predictions, requiring direct functional experimentation to verify. Some targets of circUL89 were also found to be shared between circUS12 and circUL55. These interactions implicate circUL89 in the modulation of diverse biological processes, including immune evasion, viral survival, and latency establishment. The breadth of its predicted miRNA interactions suggests that circUL89 may act as a central regulatory hub within circRNA–miRNA networks, potentially exerting widespread influence on host antiviral responses. However, despite these predictions, precise experimentation involving ectopic expression of circUL89 would be required to explore the distinct physiological effects of the transcription of the circRNA.

## 7. Differentially Expressed Host circRNAs upon HCMV Infection

Recent initiatives in HCMV circRNA research have not solely focused on the transcriptional profile of the virus during latent and lytic infection. The molecular transition between the latent and lytic stages of the virus also influences the transcriptional behavior of the host cell, which includes the expression of circRNAs corresponding to the host genome. Previous research regarding the exploration of certain host circRNAs is driven by what functions the parental genes are involved in and how the circRNA varies transcriptionally upon infection. Although the functionality of circRNAs is certainly an area that researchers aim to study, conclusions are often not definitive and merely suggest potential function based on behavior demonstrated by experimental data. However, multiple papers have suggested that the human circRNAs expressed upon HCMV infection are involved in protein scaffolding or miRNA sponging [70].

### 7.1. circSP100

One circRNA found to be upregulated when HELFs were infected with HCMV was circSP100. The parental gene of circSP100 is SP100 and is known to be associated with alternative splicing to encode a variety of protein variations. SP100 proteins are primarily localized to promyelocytic leukemia bodies (PML-MBs), which are known to be integral to transcriptional repression of viral genomes [71]. They serve as effective biomarkers for specific therapeutic courses. Exploring whether their circular counterparts could be utilized in a similar manner may provide more insight into circRNA functionality and utility. Kinetically, circSP100 levels continuously increase post-infection, whereas linear SP100 mRNA levels eventually decline [34]. Therefore, this co-transcribed non-coding molecule provides valuable context for the pathogenesis of HCMV infection.

Analysis of proteins that bind circSP100 revealed that a total of 257 proteins can bind to the circRNA, of which 10 were encoded by HCMV; localization of all circSP100-binding proteins was determined to be either cytosolic (59%) or nuclear (37%) [34]. KEGG enrichment analysis, a bioinformatic technique that identifies molecular networks that are significantly overrepresented, indicated that a considerable number of proteins were associated with the spliceosome pathway, suggesting a definitive impact on the transcriptional regulation of the host cell. Specific proteins, such as PRKDC and XRCC6/Ku70, are crucial components of the DNA-dependent protein kinase (DNA-PK) complex, which is relevant to antiviral defense mechanisms [72]. Consequently, further study of the interaction between circSP100 and the efficacy of this antiviral mechanism is needed to determine whether this upregulated circRNA can serve as a therapeutic agent against HCMV infection.

### 7.2. circMAP3K1

The circRNA, circMAP3K1, was also identified by comparing the overall transcription between mock-infected and HCMV-infected cells. Researchers selected this circRNA as a candidate for future study based on its relevance to parent gene functionality [34]. MAP3K1 is a member of a specific kinase family that regulates JNK activation, ubiquitylates c-Jun and ERK1/2, and is involved in cell migration and apoptosis [73]. Within the mechanistic framework governing the parental gene, it is posited that given the established correlation between specific MAP3K1 aberrations and diverse oncogenic phenotypes, reciprocal interactions between the exonic regions of circMAP3K1 and the linear mRNA transcript that downregulate kinase activity may result in cellular transformation. By disrupting these regulatory axes, such interactions would hypothetically promote the evasion of programmed cell death, thereby sustaining the viability of lineages otherwise designated for apoptotic clearance. However, more research is required to establish this relationship in the context of HCMV-infected cells.

### 7.3. circPLEKHM1

Both circPLEKHM1 and circTRIO were primarily selected owing to their distinct differential expression profiles following HCMV infection. Specifically, circPLEKHM1 exhibited negligible basal transcription in mock-infected cohorts, whereas authentic HCMV-infected HELFs yielded a robust accumulation of this transcript [34]. In terms of host gene functionality, PLEKHM1 is involved in the positive regulation of pathways that are necessary for endocytosis and autophagy, which are means of cellular degradation and recycling [74].

While circPLEKHM1 is strongly induced during HCMV infection in HELFs, its functional characterization to date comes primarily from non-viral cancer models. Independent studies in pancreatic ductal adenocarcinoma (PDAC) and lung cancer have shown that circPLEKHM1 can promote tumorigenesis and metastasis via interactions with FXR1 and PABPC1 [75]. Given that certain cellular targets are shared between PDAC-affecting tissues and HCMV infection-prone tissues, one may hypothesize that the upregulation of circPLEKHM1 during infection may exacerbate the progression of PDAC. Notably, circPLEKHM1 is predominantly localized within the cytoplasmic compartment. This spatial distribution is supported by prior investigations into hypoxic exosome-mediated lung cancer metastasis driven by circPLEKHM1, which utilized fluorescence in situ hybridization (FISH) and immunoprecipitation assays to demonstrate the definitive colocalization of this circRNA with PABPC1 [76]. Given the evolutionary conservation of this sequence in murine counterparts, the development of humanized mouse models offers a promising avenue to elucidate the functional interactions between this circRNA and the specific malignancies hypothesized to be driven by circPLEKHM1 upregulation [77]. However, the concept that HCMV-induced upregulation of circPLEKHM1 exerts analogous effects in the context of viral infection or contributes to viral pathobiology remains to be experimentally determined.

### 7.4. circTRIO

Compared with the transcriptional profile of circPLEKHM1 in HELFs, circTRIO was identified as the most abundant circRNA species under mock-infected conditions but became virtually undetectable following infection [34]. The parental gene, TRIO, encodes a conserved Rho guanine nucleotide exchange factor that is primarily associated with neurological development and signaling pathways [78]. Like circPLEKHM1, circTRIO was ultimately confirmed to localize predominantly in the cytoplasm through fluorescence in situ hybridization (FISH) and cytoplasmic/nuclear RNA fractionation assays, which were used to determine its intracellular distribution [79].

Non-HCMV-related studies investigating the functional role of circTRIO have demonstrated that its expression is significantly upregulated in the TU212 and TU686 cell lines, human squamous cell carcinoma models derived from head and neck squamous cell carcinoma (HNSCC) and laryngeal squamous cell carcinoma (LSCC), respectively [79]. Subsequent circTRIO knockdown experiments resulted in reduced cellular proliferation and tumorigenic capacity, indicating that circTRIO promotes the growth and progression of these cancers [79]. However, the functional significance of HCMV-mediated circTRIO depletion, and whether it represents a host antiviral response or a viral strategy to redirect host cell signaling, is currently unknown. Although further studies are needed to determine whether HCMV infection directly influences circTRIO regulation in squamous cell carcinomas, these dynamic changes in circRNA expression highlight potential mechanisms through which host circRNAs may be leveraged as novel therapeutic targets.

### 7.5. circHIPK3

Another circRNA, circHIPK3, exhibited substantial changes in expression following HCMV infection [5]. Transcriptomic analyses of HCMV-infected human embryonic lung fibroblasts (HELFs) identified circHIPK3 as a host circRNA that becomes significantly upregulated during infection, suggesting a potential role in the host–virus regulatory network [5]. CircHIPK3 is generated from exon 2 of the HIPK3 gene and is predominantly localized within the cytoplasm, where it functions primarily as a competing endogenous RNA (ceRNA) by sponging multiple microRNAs and regulating downstream gene expression [70].

In cancer biology, circHIPK3 is a well-characterized oncogenic regulator that sponges multiple microRNAs to promote cell survival across various malignancies. However, these oncogenic pathways have been defined independently of HCMV infection. Present evidence does not demonstrate that HCMV-induced circHIPK3 expression drives cellular transformation, though it highlights circHIPK3 as a responsive host factor during infection [5]. Functional studies have demonstrated that circHIPK3 participates in the regulation of certain cellular behaviors, like cellular proliferation, migration, apoptosis, and cell-cycle progression through interference with certain miRNA signaling pathways. These interactions even contribute to tumorigenic environments, as numerous studies have demonstrated their association with a multitude of malignancies. Elevated circHIPK3 expression has been reported in colorectal cancer (CRC), hepatocellular carcinoma (HCC), gastric cancer (GC), cholangiocarcinoma (CCA), gallbladder cancer (GBC), bladder cancer (BC), renal carcinoma (RC), epithelial ovarian cancer (EOC), cervical cancer (CC), prostate cancer (PCa), chronic myeloid leukemia (CML), glioma, and osteosarcoma (OS), where it frequently acts as an oncogenic regulator that promotes tumor growth, invasion, metastasis, and therapeutic resistance [80,81]. In many of these cancers, circHIPK3 exerts its effects through miRNA sequestration, thereby modulating pathways involved in cellular survival and proliferation. Despite the absence of evidence demonstrating that HCMV infection directly causes the development of certain cancers, this circRNA may possess significant biomarker potential, providing valuable insights into cancer progression and disease status.

## 8. Current Challenges and Future Directions in Functional Characterization of HCMV-Associated circRNAs

Although numerous HCMV-associated circRNAs have been identified through transcriptomic profiling, no explicit conclusions have been made regarding whether these virally encoded agents directly cause certain diseases or malignancies [5]. Recent herpesvirus circRNA studies have primarily focused on characterizing transcriptomic changes across different stages of infection by identifying differentially expressed viral and host circRNAs and predicting their potential molecular interactions to infer possible mechanisms of pathogenesis [6]. Future studies should prioritize experimental validation of these transcripts in relation to their suspected functions. By focusing on inhibiting the production of certain circRNA transcripts individually, researchers can confirm if any resulting phenotypic variation or propensity to proliferate uncontrollably can be linked to the circRNA of interest. Certain methodologies, including CRISPR-based technologies such as the Cas13 system, can selectively degrade circular RNA transcripts while minimizing disruption of their corresponding linear transcripts; similarly, precise knockdown approaches, such as RNA interference (RNAi) using the Ago2 complex to target back-splice junction (BSJ) sequences, may enable selective circRNA depletion without altering host gene expression, thereby providing more reliable models for functional characterization [52].

Alternative avenues for investigating the potential oncogenic capacities of circRNAs include the ectopic upregulation of specific candidates to elucidate the distinct relationship between virally encoded circRNAs and malignancy. While previous functional studies of circRNAs, such as circHIPK3 [82], have evaluated whether targeted downregulation or upregulation induces oncogenic phenotypes, limited research has explored the HCMV-encoded circUS12, circUL55, and circUL89. Given that the hypothesized functions of these viral circRNAs involve miRNA sponging, they hold promise as diagnostic biomarkers, provided their target miRNAs are mechanistically implicated in pathogenesis [83]. Consequently, these candidate circRNAs represent compelling targets for future investigation, particularly in deciphering the oncomodulatory effects of HCMV across various malignancies.

An additional challenge lies in the complex biogenesis of circRNAs, given that a single genomic locus can generate multiple circRNA isoforms through alternative back-splicing [84]. Consequently, disruption of a single BSJ may not eliminate circRNA expression or may affect only a subset of isoforms. Future studies should therefore incorporate isoform-specific validation methods, including divergent primer design and long-read sequencing, to accurately distinguish individual circRNA species [52]. Because HCMV infection extensively remodels host RNA processing, understanding how viral proteins regulate circRNA biogenesis will also be essential for interpreting circRNA function. Collectively, combining precise genome-editing approaches with improved methods for isoform-specific detection will provide critical insight into the biological significance of HCMV-regulated circRNAs and their potential contributions to viral replication, persistence, and disease pathogenesis.

## 9. Conclusions

Recent advances in transcriptomic sequencing and bioinformatic analyses have substantially expanded the catalog of circular RNAs associated with HCMV infection, revealing that both viral- and host-derived circRNAs are dynamically regulated throughout the viral life cycle. Collectively, current evidence suggests that these molecules may participate in diverse regulatory processes, including modulation of microRNA activity, protein interactions, immune responses, viral replication, and latency. However, many of the proposed functions remain based on differential expression analyses and computational predictions, with direct mechanistic evidence still lacking.

Continued development of circRNA-specific experimental approaches, including precise genome editing, targeted knockdown strategies, and long-read sequencing technologies, will be essential for defining the biological roles of individual HCMV circRNAs while distinguishing them from their linear transcript counterparts. These methodologies will also facilitate the investigation of the molecular mechanisms underlying circRNA biogenesis, host–virus interactions, and their contributions to HCMV persistence and pathogenesis.

As the field continues to mature, the functional characterization of HCMV-associated circRNAs may identify new targets to evaluate in future biomarker or therapeutic studies. Ultimately, integrating transcriptomic, molecular, and functional studies will provide a more comprehensive understanding of the HCMV transcriptome and may support the development of innovative antiviral strategies for HCMV-associated diseases.

## Figures and Tables

**Table 1:** Summary of researched HCMV-associated circRNAs expressed by both viral and human genomes after HCMV infection.

**CircRNA**	**Location**	**Localization**	**Expression Level After Infection**	**HCMV-Encoded or Host-Encoded**	**Evidence During HCMV Infection**	**Level of Evidence**	**Predicted Mechanism**	**Function Reported in Other Biological Contexts**	**Reference**
circUS12	US12	cytoplasm	High level of abundance (246 BSJ reads)	HCMV	Expressed during lytic infection in HELFs; BSJ verified via Sanger sequencing & RNase R resistance	Structural Validation and Computational functional prediction	Computational sponge prediction for host/viral miRNAs	Uncharacterized in non-viral models	[6]
circUL55	UL55	cytoplasm	Medium level of abundance (68 BSJ reads)	HCMV			Predicted miRNA-binding capacity		
circUL89	UL89	cytoplasm	High level of abundance (100 Junction reads)	HCMV			Predicted miRNA-binding capacity		
circSP100	SP100	Primarily cytoplasm	Upregulated upon infection	Host	Differential expression during lytic infection in HELFs w/HAN strain; Validation through RT-PCR and RT-qPCR, Sanger Sequencing, Subcellular Fractionation and Northern Blot	Interaction Experimentally Demonstrated w/Spliceosome Pathway	Hypothesized modulation of antiviral defense pathways	Interference with DNA-PK complex (inhibition of antiviral defense)	[34]
circMAP3K1	MAP3K1	cytoplasm	Upregulated upon infection	Host		Expression Change Only	Hypothesized reciprocal interactions with native gene transcripts	Downregulation of kinase activity → promotion of cellular transformation and evasion of programmed cell death	
circPLEKHM1	PLEKHM1	cytoplasm	Upregulated upon infection	Host			Sponging of RNA-binding or translation-related proteins	Promotes tumorigenesis and metastasis in non-viral cancers	
circTRIO	TRIO	cytoplasm	Downregulated upon infection	Host		Functional Phenotype Demonstrated in non-viral contexts	Predicted competitive endogenous RNA (ceRNA) network	Drives tumor growth and progression in HNSCC and LSCC	
circHIPK3	HIPK3	Nucleus, cytoplasm	Upregulated upon infection	Host	Differential expressions during lytic infection in TB40/E-infected HFFs, ECs, and NPCs and HAN-infected HELFs		Sponge for host miRNAs in non-viral contexts	Acts as oncogenic regulator that promotes tumor growth, invasion, metastasis, and therapeutic resistance	[5]

## Data Availability

No new data were created or analyzed in this study. Data sharing is not applicable to this article.
